# *Lactobacillus delbrueckii* subsp. *bulgaricus* 2038 and *Streptococcus thermophilus* 1131 ameliorate barrier dysfunction in human induced pluripotent stem cell-derived crypt-villus structural small intestine

**DOI:** 10.3389/fimmu.2025.1585007

**Published:** 2025-06-11

**Authors:** Kyosuke Kobayashi, Yuri Imai, Yuka Shimizu, Isamu Ogawa, Takaaki Nakai, Yuri Mizuno, Yoshika Kaneda, Yumiko Watanabe-Yasuoka, Fuka Yamazaki, Junko Mochizuki, Toshihiro Sashihara, Tamihide Matsunaga, Takahiro Iwao

**Affiliations:** ^1^ Wellness Science Labs, Meiji Holdings Co., Ltd., Tokyo, Japan; ^2^ Education and Research Center for Clinical Pharmacy, Faculty of Pharmaceutical Sciences, Nagoya City University, Nagoya, Japan; ^3^ Department of Clinical Pharmacy, Graduate School of Pharmaceutical Sciences, Nagoya City University, Nagoya, Japan; ^4^ Department of Molecular and Cellular Health Sciences, Graduate School of Pharmaceutical Sciences, Nagoya City University, Nagoya, Japan; ^5^ Health Science Research Unit, R&D Division, Meiji Co., Ltd., Tokyo, Japan

**Keywords:** induced pluripotent stem cell-derived crypt-villus structural small intestine, barrier function, differentiation, anti-inflammation, lactic acid bacteria, tight junction, mucin 2

## Abstract

**Background:**

Lactic acid bacteria (LAB) have been widely used as probiotics which contribute to our health. We previously reported that *Lactobacillus delbrueckii* subsp. *bulgaricus* 2038 and *Streptococcus thermophilus* 1131, two yogurt starter strains, ameliorate the intestinal barrier dysfunction caused by tumor necrosis factor (TNF)-α and interferon (IFN)-γ in Caco-2 cells. However, Caco-2 cells differ from living organisms in various ways. We have developed a human induced pluripotent stem cell-derived crypt-villus structural small intestine (hiPSC-SI) was established with a villus-like structure containing constituent cells of the small intestine.

**Methods:**

A hiPSC-SI and LAB co-culture model was established to assess the impact of LAB on barrier function and elucidate the underlying mechanisms.

**Results:**

The medium on the luminal side for co-culturing cells and bacteria was examined and determined to use Hanks’ balanced salt solution without glucose in terms of bacterial survival rate. LAB were found to ameliorate permeability and decrease the gene expression of tight junction associated proteins induced by TNF-α and IFN-γ. Regarding cell differentiation, LAB suppressed the downregulation of *LGR5*, *VIL1*, *LYZ* and *MUC2* by cytokines. Moreover, they ameliorated reduced mucin 2 protein production and decreased the number of mucin 2-positive cells. Finally, transcriptome analysis suggested that they ameliorated the aberration in cytokine-induced cell differentiation via an anti-inflammatory effect on intestinal stem cells.

**Conclusions:**

The results indicate that LAB ameliorate the cytokine-induced dysfunction of intestinal barrier integrity and homeostasis disrupted by cytokines in a co-culture model of hiPSC-SI and LAB.

## 1 Introduction

The small intestine is a key organ for the absorption of nutrients from food and water (1). However, as a result of this role, it is constantly exposed to foreign substances. This highlights the importance of regulating intestinal integrity, including intestinal barrier function and homeostasis. Tight junctions (TJ) are physical barriers that prevent foreign substances from passing into the body by tightly sealing the intercellular space of epithelial cells (2). Homeostasis is maintained by a range of cells, including goblet and Paneth cells, which produce mucus and antimicrobial peptides, respectively (3). Each cell plays an important role in forming the epithelium of the small intestine. Intestinal stem cells (ISCs) play a central role in the proliferation and differentiation of small intestinal epithelial cells (IECs).

Lactic acid bacteria (LAB) are widely used as probiotics to support intestinal health. *Lactobacillus delbrueckii* subsp. *bulgaricus* 2038 and *Streptococcus thermophilus* 1131 are two commonly used starter strains for yogurt production. Both strains are known to induce the expression of antimicrobial peptides in the small intestines of mice by stimulating immune cells (4). Moreover, in *in vitro* experiments using Caco-2 cells, both strains were found to directly stimulate the cells and ameliorate the physical barrier dysfunction caused by tumor necrosis factor (TNF)-α and interferon (IFN)-γ (5).

Animal experiments are often conducted to estimate the beneficial effects of LAB on the intestines of living organisms. However, experimental methods focusing on improving animal welfare are currently lacking. Although Caco-2 cells, which are derived from a colon carcinoma and differentiate into enterocytes with brush border found in the small intestine, have been widely used for the study of small IECs, issues such as electrical resistance and the expression of transporters and metabolic enzymes, which significantly deviate from the conditions in living organisms, have been reported (6, 7). Consequently, small intestinal organoids that are 3D cultures of small intestinal crypts were developed (8). Small intestinal organoids can also be produced from human induced pluripotent stem cells (hiPSC) using appropriate differentiation methods (9). However, as the luminal side faces the inside of small intestinal organoids, microinjections are required to evaluate the function of food materials. To address this, an experimental technique was developed to cultivate small intestinal organoids in a monolayer by seeding them on a flat surface after single-cell enzymatic treatment, thereby simplifying their handling (10, 11). Furthermore, it is now possible to obtain a monolayer of small IECs by continuously differentiating hiPSCs on flat surface (12–14). Although these methods produce two-dimensional (2D) monolayer sheets that are easy to use, they lack the crypt and villi, two fundamental structures of the small intestine in living organisms. 2D monolayer sheets are composed of various cells arranged randomly. In living organisms, Paneth cells produce niche signals for stem cells at crypt bottoms (15), and most enterocytes and goblet cells are located in villi (16). This suggests that the response of 2D monolayer sheets to stimulation will be inherently different from that of living organisms. To address this problem, we previously developed a hiPSC-derived crypt-villus structural small intestine (hiPSC-SI), which has a villi-like structure and contains constituent cells of the small intestine, including stem, Paneth, and goblet cells (17, 18). The hiPSC-SI is similar to the human living organism regarding barrier function. The trans-epithelial electrical resistance (TEER) value of hiPSC-SI is 150–200 Ω·cm^2^ (18), which is similar to that of the human small intestine (50–100 Ω·cm^2^) (19). This *in vitro* model represents an important alternative to animal experiments.

In the present study, a co-culture model of hiPSC-SI was established using *L*. *bulgaricus* 2038 and *S. thermophilus* 1131 to assess the impact of LAB on barrier function. The effects of both strains on intestinal barrier function were evaluated using this model. Taking advantage of the characteristics of hiPSC-SI cells, we also explored the effects of both strains on cell differentiation.

## 2 Materials and methods

### 2.1 hiPSC-SI preparation

hiPSCs (#51: Windy) are human ES cell-like colonies cloned from human embryonic lung fibroblasts (MRC-5) after the transduction of *OCT3/4*, *SOX2*, *KLF4*, and *c-MYC* with a pancreatic retroviral vector. Cells were kindly provided by Dr. Akihiro Umezawa (National Center for Child Health and Development, Tokyo, Japan) and cultured as previously reported (20).

hiPSCs were differentiated into intestinal organoids and cultured on cell culture inserts as previously reported (17). hiPSCs were seeded onto a culture dish coated with Dulbecco’s modified Eagle medium/F12 (DMEM/F12; Wako, Osaka, Japan) containing 20% KnockOut serum replacement (Thermo Fisher Scientific, Waltham, MA, USA), 0.08 mM non-essential amino acids (Biological Industries, Beit-Haemek, Israel), 2 mM L-Gln, 0.1 mM 2-mercaptoethanol and 3% Matrigel (Becton Dickinson, Cockeysville, MD, USA). The cells were cultured in StemSure hPSC medium (Wako) containing 35 ng/mL fibroblast growth factor 2 (FGF2; PeproTech, Rocky Hill, NJ, USA). When the confluency of the cells was 80-90%, the cells were cultured with Roswell Park Memorial Institute (RPMI) 1640 medium (Thermo Fisher Scientific) containing 100 ng/mL activin A (PeproTech), 100 units/mL penicillin G, 100 μg/mL streptomycin and 2 mM L-Glu for 24 h. Next, 0.2% fetal bovine serum (FBS) (Nichirei Biosciences, Tokyo, Japan) was added, and the cells were cultured for 24 h, followed by the addition of 2% FBS for 24 h, leading to their differentiation into endoderm. The cells were then differentiated into mid- and hindgut-like cells by culturing them in RPMI 1640 medium containing 1% GlutaMAX (Gibco, Carlsbad, CA, USA), 2% FBS, 3 µM CHIR99021, 500 ng/mL FGF4 (BioLegend, San Diego, CA, USA), 100 units/mL penicillin G, and 100 µg/mL streptomycin for 96 h. Mid- and hindgut-like cells were treated with Accutase (MS Technosystems, Osaka, Japan) for 5 min and single-celled by pipetting after collection in centrifuge tubes in Advanced-DMEM/F12 (Thermo Fisher Scientific) medium containing 10% FBS. Cells (2.0 × 10^5^) were then seeded onto 10 cm^2^ dishes coated with iMatrix-511 silk (Nippi, Tokyo, Japan). Fresh medium of Advanced-DMEM/F12 containing 2 mM GlutaMAX supplemented with 2% B27 (Thermo Fisher Scientific) and 1% N2 (Thermo Fisher Scientific) was added to the cells, along with 100 units/mL penicillin G, 100 µg/mL streptomycin (differentiation medium (DM)) with 2% FBS, 100 ng/mL epidermal growth factor (EGF) (GenScript, Piscataway, NJ, USA), 10 µM Y-27632 (Focus Biomolecules, Plymouth Meeting, PA, USA), 0.5 µM A-83-01 (AdooQ Bioscience, Irvine, CA, USA), 3 µM CHIR99021 (Focus Biomolecules), and 30 ng/mL FGF2 (Peprotech). The cells were filtered using a 40-µm nylon-mesh cell strainer (Becton Dickinson) before seeding 4.0 × 10^6^ cells on 100-mm EZSPHERE (AGC Techoglass, Shizuoka, Japan) and culturing for 3 d in DM containing 100 ng/mL EGF (GenScript), 100 ng/mL Noggin (GenScript), 200 ng/mL R-spondin 1 (GenScript), and 10 µM Y-27632 (Focus Biomolecules). Next, the cells were suspended in DM containing 3% Matrigel as a culture substrate for nine days to differentiate into intestinal organs. The medium was changed every 3 d.

**Figure 1:**
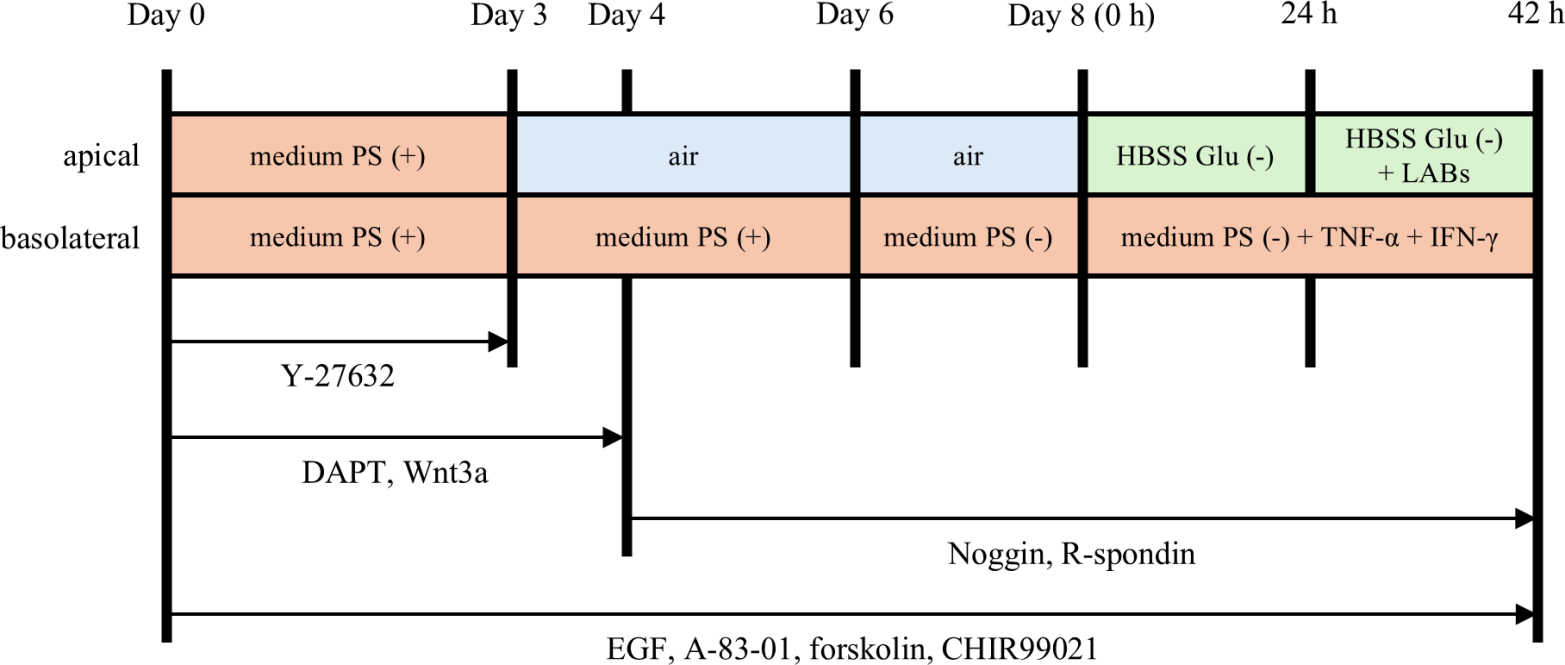
Culture and experimental protocol for human induced pluripotent stem cell-derived crypt-villus structural small intestine (hiPSC-SI). hiPSC-derived small intestinal organoids were dispersed as single cells and seeded onto ThinCert 24 well cell culture inserts. The cells were cultured in conditioned medium containing various differentiation factors. From day 3, the cells were cultured at the air-liquid interface. From day 8 onwards, the activity of lactic acid bacteria (LAB) on hiPSC-SI was examined. PS, penicillin and streptomycin; Glu, glucose.

The hiPSC-SI model was prepared from organoids as previously reported (17, 18). Briefly, the organoids were treated with TrypLE Select (Thermo Fisher Scientific) at 37°C for 15 m and centrifuged at 200×g for 5 m. The cells were suspended in Advanced-DMEM/F12 containing 2% FBS, 2 mM GlutaMAX, 15 mM HEPES, 2% B27, 1% N2 (conditioned medium) with 100 units/mL penicillin G, 100 µg/mL streptomycin and additional supplements as shown in **Figure 1** and seeded onto ThinCert 24-well cell culture inserts with a pore size of 1.0 µm (Greiner Bio-One, Kremsmünster, Austria) coated with iMatrix-511 silk (Nippi). The cells were cultured at the air-liquid interface and co-cultured with bacteria as described in **Figure 1** based on the experiments in the **Supplementary Materials**.

### 2.2 LAB culture

*L. bulgaricus* 2038 and *S. thermophilus* 1131 were cultured anaerobically with AnaeroPouch-Anaero (Mitsubishi Gas Chemical, Tokyo, Japan) at 37°C for 18 h in de Man Rogosa Sharpe (MRS) broth (Becton Dickinson) and M17 broth (Becton Dickinson) supplemented with 1% lactose (LM17), respectively. LAB were washed twice with phosphate-buffered saline (PBS; pH 7.4) and resuspended in PBS until an optical density at 600 nm (OD_600_) of 10.0 using a BioTek SYNERGY HTX multi-mode reader (Agilent, Tokyo, Japan) and UVmini-1240 (SHIMADZU, Kyoto, Japan).

### 2.3 Screening of media for LAB co-culture with hiPSC-SI

*L. bulgaricus* 2038 and *S. thermophilus* 1131 were suspended in conditioned medium with or without penicillin and streptomycin (PS (+) or PS (–)) or Hanks’ balanced salt solution (HBSS) with or without glucose (Glu (+) or Glu (–)) was as the OD_600_ to be 0.1 and cultured anaerobically at 37°C for 18 h. Serial dilutions of the LAB culture were plated onto MRS or LM17 agar plates and cultured under anaerobic conditions at 37°C for 24 h. Subsequently, colony-forming units (CFU) were counted.

On co-cultivation with hiPSC-SI, LAB were suspended in HBSS Glu (–) so that OD_600_ was 0.1 and cultured at 37°C and 5% CO_2_ for 18 h. Then, the culture was collected, and the viability of both strains was evaluated as mentioned above.

### 2.4 Experimental design

A schematic representation of the experimental schedule is provided in **Figure 1**. On day 8 of the hiPSC-SI culture, the medium on the apical side was replaced with HBSS Glu (–). TNF-α and IFN-γ are reported to increase barrier permeability of IECs (21, 22). For the barrier destruction group, TNF-α (10 ng/mL; R&D Systems, Minneapolis, MN, USA) and IFN-γ (10 ng/mL; R&D Systems) were added to the basolateral side and incubated for 42 h. The concentration of cytokines was referred to our previous report using Caco-2 cells (5). To examine the activities of LAB against the stimulation of TNF-α and IFN-γ, live LAB were added to the apical side as the OD_600_ to be 0.1, 24 h after the cytokines were added to the basolateral side. After co-culture for 18 h, intestinal barrier function was assessed. hiPSC-SIs were collected for analysis of gene expression, mucin 2 levels, and immunofluorescence.

### 2.5 Measuring the intestinal barrier function

TEER value was measured using an EVOM3 Epithelial Volt/Ohm Meter (World Precision Instruments, Sarasota, FL, USA). To assess the permeability of the cell monolayers, fluorescein isothiocyanate-dextran with an average molecular weight of 4,000 Da (FD-4; Sigma-Aldrich, St Louis, MO, USA) was used. The medium on the basolateral side was replaced with HBSS containing 10 mM HEPES. The apical side was replaced with the same buffer containing 1 mg/mL FD-4. The medium on the basolateral side was collected after 30 min, and the fluorescence emission at 528 nm was measured after excitation at 420 nm using a Synergy HTX multi-mode plate reader and Gen 5 data analysis software (BioTek, Santa Clara, CA, USA). The apparent permeability coefficient (P*_app_*) was calculated using the following equation:


$$\text{P}app=\frac{\text{C}\times \text{V}}{\text{Cini}\times \text{t}\times \text{S}}$$

where C is the amount of FD-4 that permeated, V is the volume of the buffer on the basolateral side, C_ini_ is the initial FD-4 concentration on the apical side, t is the time of the permeability test, and S is the membrane surface area.

### 2.6 RNA isolation and real-time polymerase chain reaction (PCR)

Total RNA from hiPSC-SI was isolated using the Maxwell RSC simpleRNA Cells Kit (Promega, Madison, WI, USA), according to the manufacturer’s protocols. Total RNA was quantified using a NanoDrop 8000 spectrophotometer (Thermo Fisher Scientific). Complementary DNA was synthesized from 1 μg of total RNA using a PrimeScript RT Master Mix (Takara Bio, Shiga, Japan), and real-time PCR was performed using a QuantStudio 3 Real-Time PCR System (Thermo Fisher Scientific) and SYBR Premix Ex Taq II (Takara Bio), according to the manufacturer’s protocol. The nucleotide sequences of the primers used are listed in **Table 1**. Quantitative comparisons were performed using the ΔΔC_T_ method. Data were normalized to the values of *HPRT1* and the results were expressed as fold changes to threshold cycle values relative to the controls.

**Table 1:** The sequence of primers for real-time polymerase-chain reaction.

Gene	Protein	Direction	Sequence (5’→3’)
*CLDN1*	claudin-1	forward	GTGCGATATTTCTTCTTGCAGG
		reverse	TTCGTACCTGGCATTGACTGG
*CLDN3*	claudin-3	forward	CTGCTCTGCTGCTCGTGTCC
		reverse	TTAGACGTAGTCCTTGCGGTCGTAG
*CLDN4*	claudin-4	forward	GGCTGCTTTGCTGCAACTGTC
		reverse	GAGCCGTGGCACCTTACACG
*CLDN7*	claudin-7	forward	TTTTCATCGTGGCAGGTCTTG
		reverse	CCCTGCCCAGCCAATAAAGA
*CLDN12*	claudin-12	forward	CTCCCCATCTATCTGG
		reverse	GGTGGATGGGAGTACA
*OCLN*	occludin	forward	CACACAGGACGTGCCTTCA
		reverse	GCTGCCTGAAGTCATCCACA
*TJP1*	ZO-1	forward	GCCAGGAAGTTATACGAGCGA
		reverse	TGGAGCTGACAGGTAGGACA
*TJP2*	ZO-2	forward	ATGGAAGAGCTGATATGGGAACA
		reverse	TGCTGAACTGCAAACGAATGAA
*TJP3*	ZO-3	forward	GCTTTGGCATTGCGATCTCTG
		reverse	GATGTGGTCGCCTGTCTGTAG
*F11R*	JAM-A	forward	ATGGGGACAAAGGCGC
		reverse	CAATGCCAGGGAGCAC
*LGR5*	leucine-rich repeat-containing G-protein-coupled receptor 5	forward	GCTTCCTGGAGGAGTTACGTC
		reverse	AGCTGATGTGGTTAGCATCCAG
*VIL1*	villin 1	forward	AGCCAGATCACTGCTGAGGT
		reverse	TGGACAGGTGTTCCTCCTTC
*MUC2*	mucin 2	forward	AGAAGGCACCGTATATGACGAC
		reverse	CAGCGTTACAGACACACTGCTC
*LYZ*	lysozyme	forward	TCAATAGCCGCTACTGGTGT
		reverse	AATGCCTTGTGGATCACGGA
*GP2*	glycoprotein 2	forward	CAATGTGCCTACCCACTGGA
		reverse	AGCACGGACTCAACAGACAG
*DCLK1*	doublecortin-like kinase 1	forward	TAGCACAGCAGCTGGAGTTT
		reverse	GAGTTGAGTTCGGGAGGAGC
*MKI67*	marker of proliferation Ki-67	forward	GACTTTGGGTGCGACTTGAC
		reverse	ACCCCGCTCCTTTTGATAGT
*CDH1*	cadherin 1	forward	ATGAGTGTCCCCCGGTATCT
		reverse	GGTCAGTATCAGCCGCTTTC
*HPRT1*	hypoxanthine phosphoribosyltransferase 1	forward	CTTTGCTTTCCTTGGTCAGG
		reverse	TCAAGGGCATATCCTACAACA

### 2.7 Microarray analysis

The total RNA concentration was determined using an Agilent Bioanalyzer 2100 and RNA 6000 Nano LabChip Kit (Agilent Technologies, Palo Alto, CA, USA). Equivalent amounts of RNA were pooled from each group. A GeneChip WT PLUS Reagent Kit (Thermo Fisher Scientific) was used to prepare the microarray samples according to the manufacturer’s protocol. The samples were hybridized to Clariom S Arrays for Humans (Thermo Fisher Scientific). All arrays were scanned using the Affymetrix GeneChip Command Console installed on a GeneChip Scanner 3000-7G. The array datasets were normalized using the signal space transformation-robust multi-chip analysis algorithm implemented in Affymetrix’s transcriptome analysis console software (version 4.0).

Pathway analysis was performed using MetaCore software (accessed on December 5, 2023) (Clarivate Analytics, London, UK), and gene set enrichment analysis (GSEA) (Broad Institute, Cambridge, MA, USA) (23, 24) was performed. For MetaCore software, genes with differential expression levels of |log_2_ fold change| > 0.5 were extracted as differentially expressed genes (DEGs) and used for pathway analysis using GSEA software (version 4.3.2) based on hallmark, Biocarta, Reactome, and Gene Ontology (GO). To compute the nominal enrichment score (NES), the permutation value was set to 1,000. Gene sets with a false discovery rate (FDR) *q*-value< 0.25 were recognized as significantly enriched. *P*< 0.05 was used to select the enriched gene sets when FDR *q*-value > 0.25.

### 2.8 Enzyme-linked immunosorbent assay for mucin 2

Cells were lysed using RLT lysis buffer (Qiagen, Venlo, Netherlands). The mucin 2 concentration in both samples was measured using the Human MUC2 (Mucin 2) ELISA Kit (MyBioSource, San Diego, CA, USA).

### 2.9 Immunofluorescence microscopy

Cells were fixed in methanol at –30°C, embedded in paraffin, and sectioned at a thickness of 4 µm. Samples were deparaffinized using Hemo-De (Falma, Tokyo, Japan) and hydrophilized using ethanol. After washing the slides with PBS, PBS containing 0.2% Triton X-100 was added, and the cells were incubated for 5 min at room temperature (RT). One drop of Image-iT FX Signal Enhancer (Thermo Fisher Scientific) was added and incubated for 30 min at RT. The cells were blocked with 2% normal goat serum for 10 min at RT and labeled with anti-mucin 2 (sc-515032; Santa Cruz Biotechnology, Santa Cruz, CA, USA) antibody for 2 h at RT, followed by incubation for 1 h at RT with fluorescein-conjugated goat anti-IgG (ab150015; Abcam, Cambridge, MA, USA). The cells were visualized using a confocal laser scanning microscope (LSM880; Zeiss, Oberkochen, Germany).

### 2.10 Statistical analysis

Data are presented as the mean ± standard error. Statistical analyses were performed using Dunnett’s test for parametric data sets or the Brunner-Munzel test followed by the Benjamini-Hochberg correction for nonparametric data sets. Statistical significance was set at *P<* 0.05.

## 3 Results

### 3.1 Selection of suitable medium for LAB co-culture with hiPSC-SI

A suitable medium is needed to appropriately evaluate the function of live bacteria in a co-culture model since bacterial growth, which is dependent on the medium, influences the experiment. Prior to examining the effect of LAB activity on hiPSC-SIs, media were evaluated for use in the co-culture of LAB and hiPSC-SIs in terms of the survival rate of LAB.

**Figure 2:**
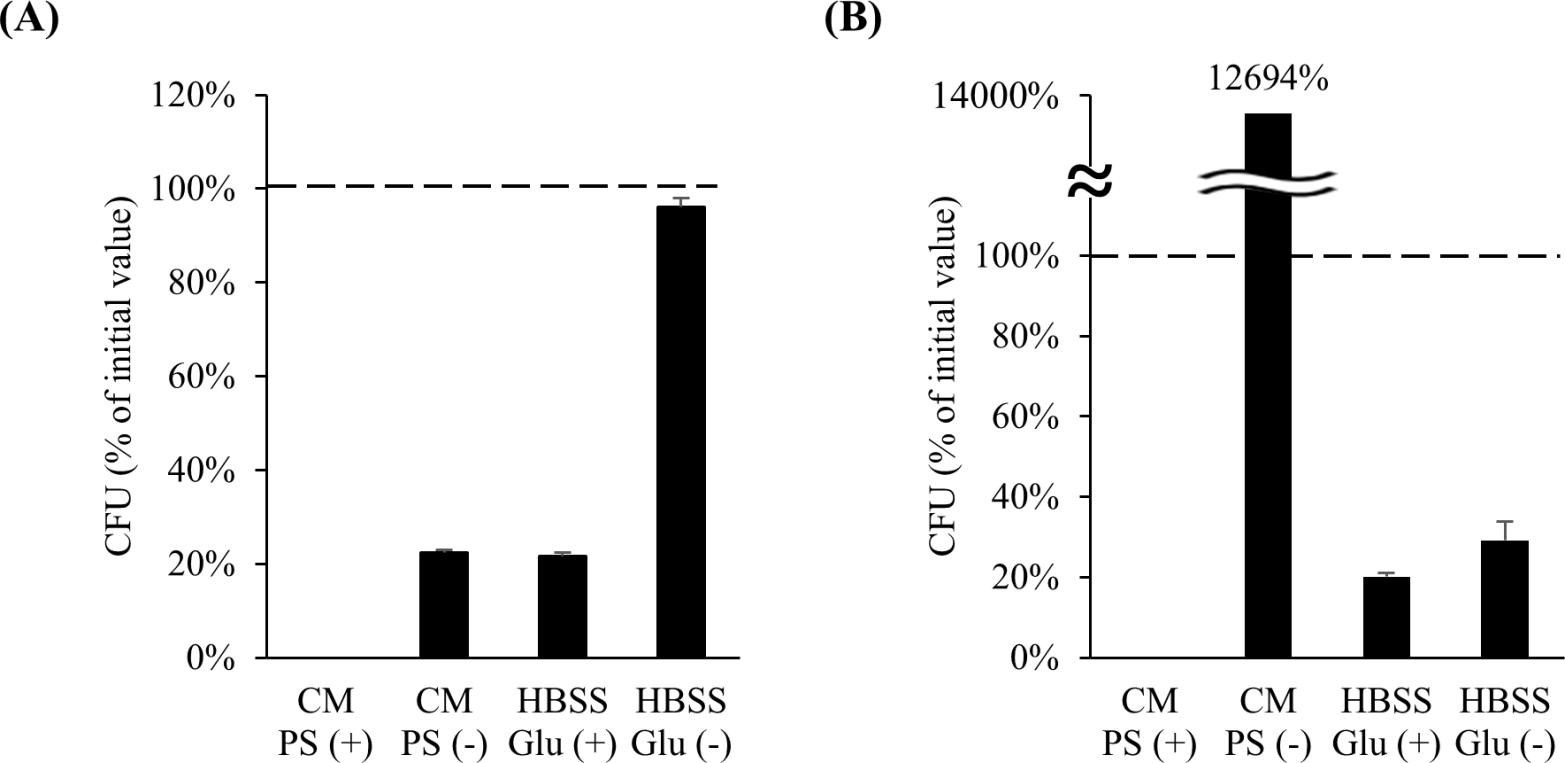
The number of *Lactobacillus delbrueckii* subsp. *bulgaricus* 2038 and *Streptococcus thermophilus* 1131 was maintained after culturing in HBSS without glucose. **(A)** *L. bulgaricus* 2038 and **(B)** *S. thermophilus* 1131 were cultured in conditioned medium with or without penicillin and streptomycin (PS (+) or PS (–)) or HBSS with or without glucose (Glu (+) or Glu (–)) anaerobically at 37 °C for 18 h. The colony-forming unit (CFU) percentage of the initial value is shown (n = 6). The dashed line represents 100%. CM, conditioned medium.

First, the conditioned media PS (+) and PS (–) were examined. LAB were not detected when cultured in the PS (+)-conditioned medium (**Figure 2**). This was expected because LAB are susceptible to PS. The cell number of *L. bulgaricus* 2038 was decreased when cultured in the PS (–)-conditioned medium (**Figure 2A**). In this case, the pH did not decrease, as there was no change in the color of the medium, suggesting that the bacteria did not grow. *S. thermophilus* 1131 substantially grew when cultured in the PS (–)-conditioned medium (**Figure 2B**). Therefore, to prevent the bacterium from overgrowing during co-cultivation, HBSS Glu (+) and HBSS Glu (–) were examined. When HBSS Glu (+) was used, the survival rates of both strains decreased (**Figure 2**). However, when cultured in HBSS Glu (–), the cell number of *L. bulgaricus* 2038 remained the same as that before culturing, whereas the cell number of *S. thermophilus* 1131 was slightly increased compared to that when cultured in HBSS Glu (+) (**Figure 2**). Therefore, we concluded that HBSS Glu (–) could be used for culturing LAB.

### 3.2 Effect of HBSS addition to the apical side on hiPSC-SI

**Figure 3:**
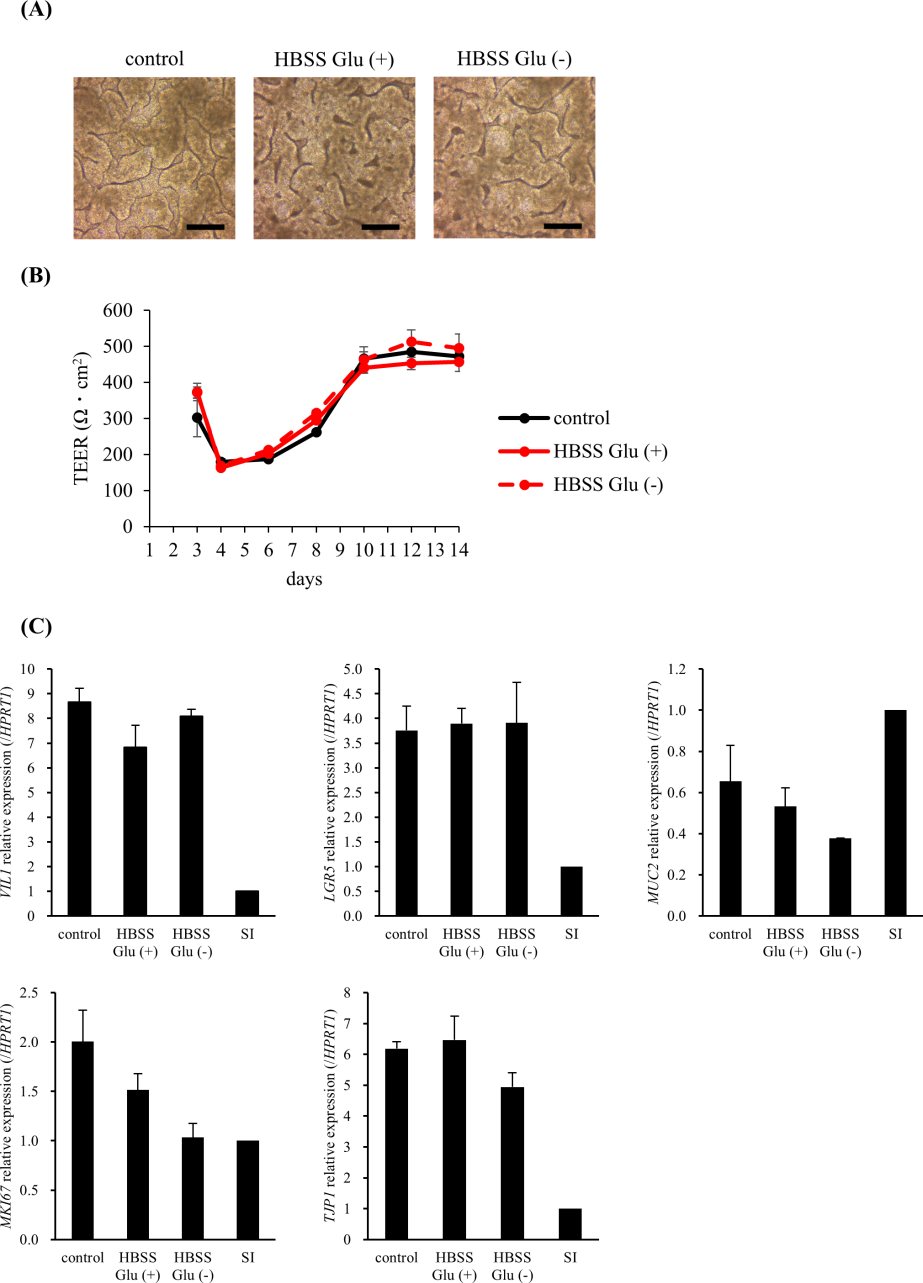
HBSS did not change the morphology, barrier function, or intestinal-associated gene expression. In the HBSS Glu (+) and HBSS Glu (–) groups, HBSS with or without glucose was added to the apical side on day 8, respectively. **(A)** The 3D structure was observed on day 14 (scale bar, 200 µm). **(B)** The trans-epithelial electrical resistance (TEER) values were measured immediately before medium change. **(C)** Cells were harvested on day 14 and intestine-associated gene expression was analyzed using real-time polymerase chain reaction. Data were normalized to hypoxanthine phosphoribosyltransferase 1 (*HPRT1*) expression and expressed relative to the adult small intestine as 1. Data are presented as mean ± standard error (n = 3).

Next, we considered a usable medium for the co-culture of cells and LAB. The medium used for hiPSC-SI caused bacterial overgrowth, which affected the condition of the cells (data not shown). HBSS with or without glucose was examined as the medium on the apical side, and no significant changes in the 3D structure, TEER value, or intestine-associated gene expression were observed, indicating that HBSS with or without glucose can be used as a co-culture medium (**Figure 3**).

### 3.3 Effect of LAB activity on intestinal barrier function

**Figure 4:**
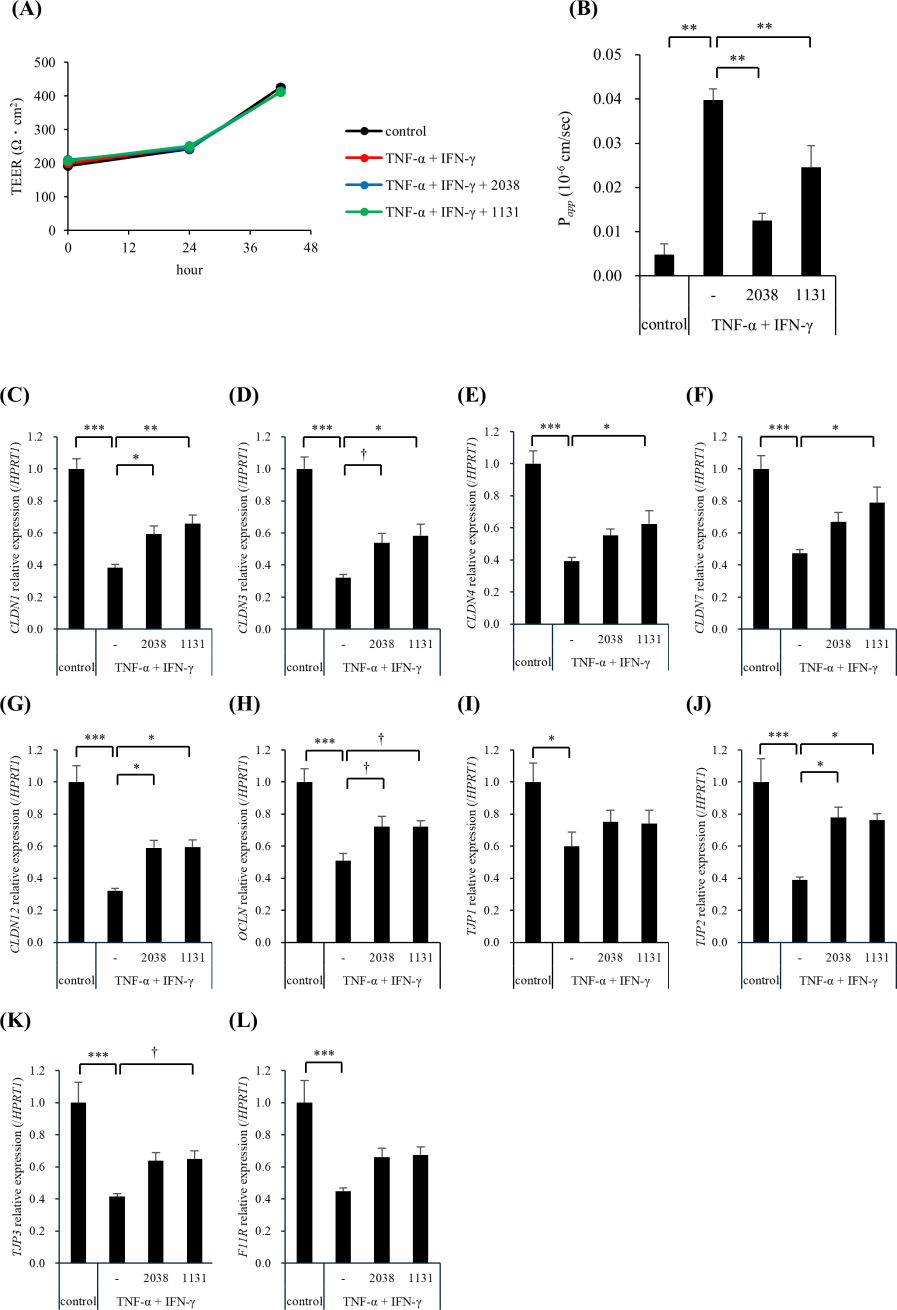
*Lactobacillus delbrueckii* subsp. *bulgaricus* 2038 and *Streptococcus thermophilus* 1131 improved the intestinal barrier dysfunction induced by TNF-α and IFN-γ*. L. bulgaricus* 2038, *S. thermophilus* 1131, TNF-α, and IFN-γ were added to hiPSC-SI on day 8. **(A)** The trans-epithelial electrical resistance (TEER) percentage of the initial value and **(B)** the permeability of fluorescein isothiocyanate-dextran with an average molecular weight of 4,000 (FD-4) after 42 h in the experiments are shown (n = 5). **(C-L)** The gene expression levels of TJ-associated proteins (**(C)** *CLDN1*, **(D)** *CLDN3*, **(E)** *CLDN4*, **(F)** *CLDN7*, **(G)** *CLDN12*, **(H)** *OCLN*, **(I)** *TJP1*, **(J)** *TJP2*, **(K)** *TJP3*, and **(L)** *F11R*) were evaluated via real-time polymerase chain reaction. Data were normalized to hypoxanthine phosphoribosyltransferase 1 (*HPRT1*) expression and are shown as relative expression levels (n = 5). Comparisons were performed using Dunnett’s test. *** *P*< 0.001, ** *P*< 0.01, * *P*< 0.05, ^†^ *P*< 0.1.

Next, we examined the effects of *L. bulgaricus* 2038 and *S. thermophilus* 1131 on the intestinal barrier function. TEER value in all groups was approximately 200 Ω·cm^2^ at 0 h when TNF-α and IFN-γ were added, gradually increasing to 250 Ω·cm^2^ at 24 h and over 400 Ω·cm^2^ at 40 h, approximately (**Figure 4A**). The stimulation of hiPSC-SI by TNF-α and IFN-γ alone and with the additional treatment of both strains did not exhibit differences at 24 and 42 h when compared with that in the control group (**Figure 4A**). On the other hand, stimulation by TNF-α and IFN-γ significantly enhanced FD-4 permeability, with both strains significantly suppressing this (**Figure 4B**).

To elucidate the underlying mechanism, we analyzed the gene expression levels of TJ-associated proteins using real-time PCR. TNF-α and IFN-γ significantly reduced the gene expression of all the examined genes compared with that in the control group (**Figures 4C–L**). *L*. *bulgaricus* 2038 significantly increased *CLDN1*, *CLDN12*, and *TJP2* expression (**Figures 4C,G,J**) and tended to increase *CLDN3* and *OCLN* expression compared with that in the TNF-α + IFN-γ group (**Figures 4D,H**). *S. thermophilus* 1131 significantly increased *CLDN1*, *CLDN3, CLDN4*, *CLDN7*, *CLDN12*, and *TJP2* expression (**Figures 4C–G,J**) and tended to increase *OCLN* and *TJP3* expression compared with that in the TNF-α + IFN-γ group (**Figures 4H,K**).

### 3.4 Effect of LAB activity on intestine differentiation

**Figure 5:**
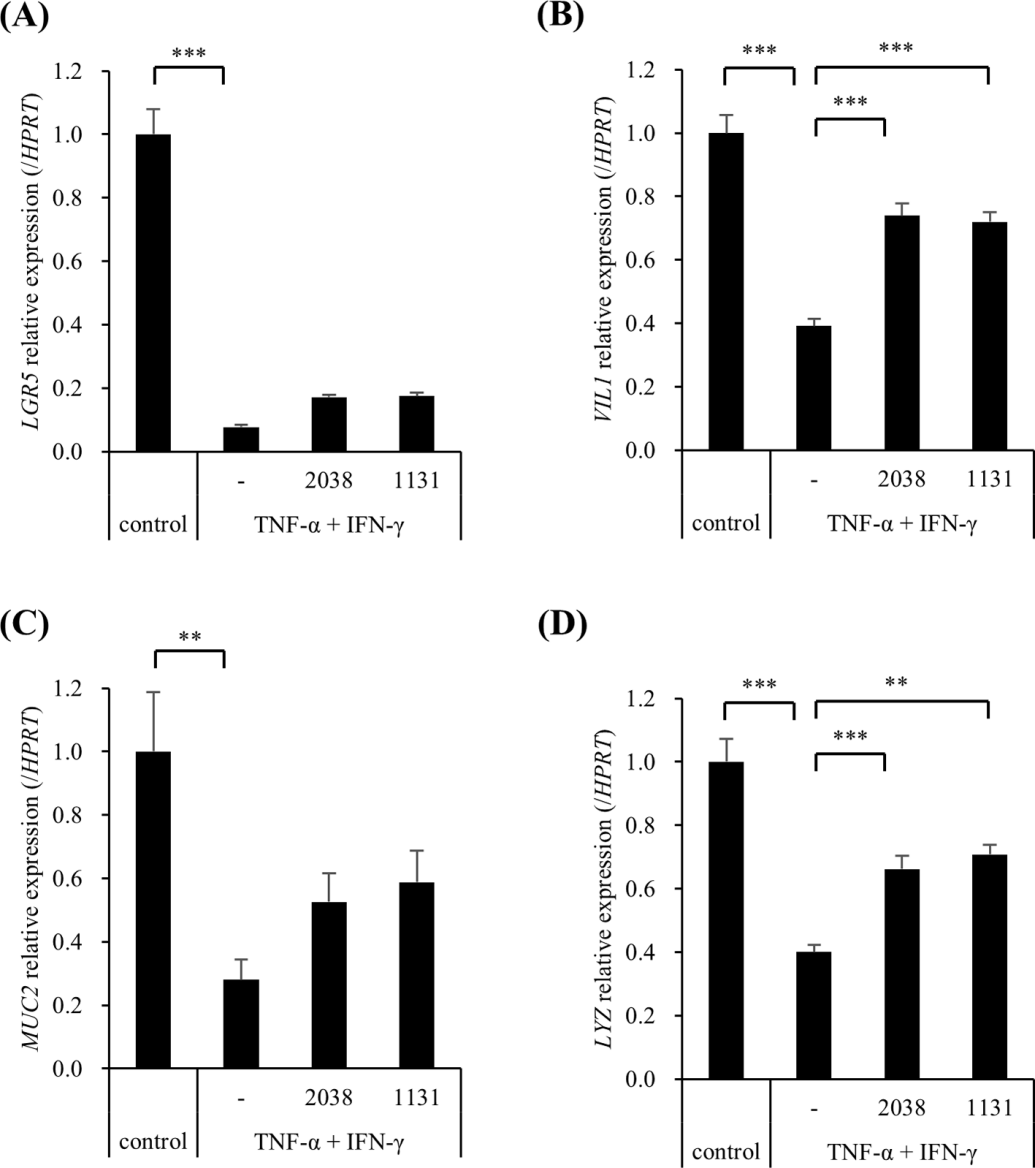
*Lactobacillus delbrueckii* subsp. *bulgaricus* 2038 and *Streptococcus thermophilus* 1131 suppressed the gene expression downregulation of cell markers by TNF-α and IFN-γ*. L. bulgaricus* 2038, *S. thermophilus* 1131, TNF-α, and IFN-γ were added to the cells. Cells were collected after 42 h in the experiments. The gene expression levels of cell markers (**(A)** *LGR5*, **(B)** *VIL1*, **(C)** *MUC2*, and **(D)** *LYZ*) were evaluated via real-time polymerase-chain reaction. Data were normalized to hypoxanthine phosphoribosyltransferase 1 (*HPRT1*) expression and are shown as relative expression levels (n = 5). Comparisons were performed using Dunnett’s test. *** *P*< 0.001, ** *P*< 0.01.

The effects of *L. bulgaricus* 2038 and *S. thermophilus* 1131 on the differentiation of hiPSC-SIs were examined using real-time PCR. TNF-α and IFN-γ significantly decreased *LGR5* (stem cell), *VIL1* (enterocyte), *MUC2* (goblet cell), and *LYZ* (Paneth cell) compared with that in the control group (**Figure 5**). Contrastingly, both strains significantly increased *VIL1* and *LYZ* expression compared with that in the TNF-α + IFN-γ group (**Figures 5B,D**). Although both strains also increased the expression of *LGR5* and *MUC2*, significant differences were not observed compared with those in the TNF-α + IFN-γ group (**Figures 5A,C**).

To elucidate the mechanism underlying LAB activity on differentiation, microarray transcriptome analysis was performed. For pathway analysis using MetaCore, genes with |log_2_ fold change| > 0.5 were extracted and pathway analysis was performed using DEGs. The DEGs were enriched in the gene sets described in **Table 2**. The results suggested that both strains activated the Wnt/β-catenin signaling pathway (**Table 2**).

**Table 2:** Metacore analysis.

Pathway	Comparison	*P* value	FDR
Immune response_Induction of apoptosis and inhibition of proliferation mediated by IFN-gamma	control vs TNF-α+IFN-γ	3.082.E-09	1.815.E-06
	TNF-α+IFN-γ vs TNF-α+IFN-γ+2038	2.090.E-03	1.981.E-02
	TNF-α+IFN-γ vs TNF-α+IFN-γ+1131	9.757.E-07	2.976.E-04
Apoptosis and survival_Regulation of apoptosis by mitochondrial proteins	control vs TNF-α+IFN-γ	4.427.E-05	1.442.E-03
	TNF-α+IFN-γ vs TNF-α+IFN-γ+2038	6.431.E-05	1.402.E-03
	TNF-α+IFN-γ vs TNF-α+IFN-γ+1131	6.215.E-05	7.898.E-04
Apoptosis and survival_Caspase cascade	control vs TNF-α+IFN-γ	2.002.E-06	1.703.E-04
	TNF-α+IFN-γ vs TNF-α+IFN-γ+2038	9.525.E-05	2.899.E-03
	TNF-α+IFN-γ vs TNF-α+IFN-γ+1131	8.917.E-04	1.600.E-02
Oxidative stress_ROS signaling	control vs TNF-α+IFN-γ	1.602.E-03	1.232.E-02
	TNF-α+IFN-γ vs TNF-α+IFN-γ+2038	5.779.E-05	2.321.E-03
	TNF-α+IFN-γ vs TNF-α+IFN-γ+1131	1.478.E-02	8.424.E-02
Development_Positive regulation of WNT/Beta-catenin signaling at the receptor level	control vs TNF-α+IFN-γ	3.110.E-07	6.802.E-05
	TNF-α+IFN-γ vs TNF-α+IFN-γ+2038	9.689.E-05	2.899.E-03
	TNF-α+IFN-γ vs TNF-α+IFN-γ+1131	1.077.E-02	7.192.E-02

### 3.5 Effect of LAB activity on mucin 2 production

**Figure 6:**
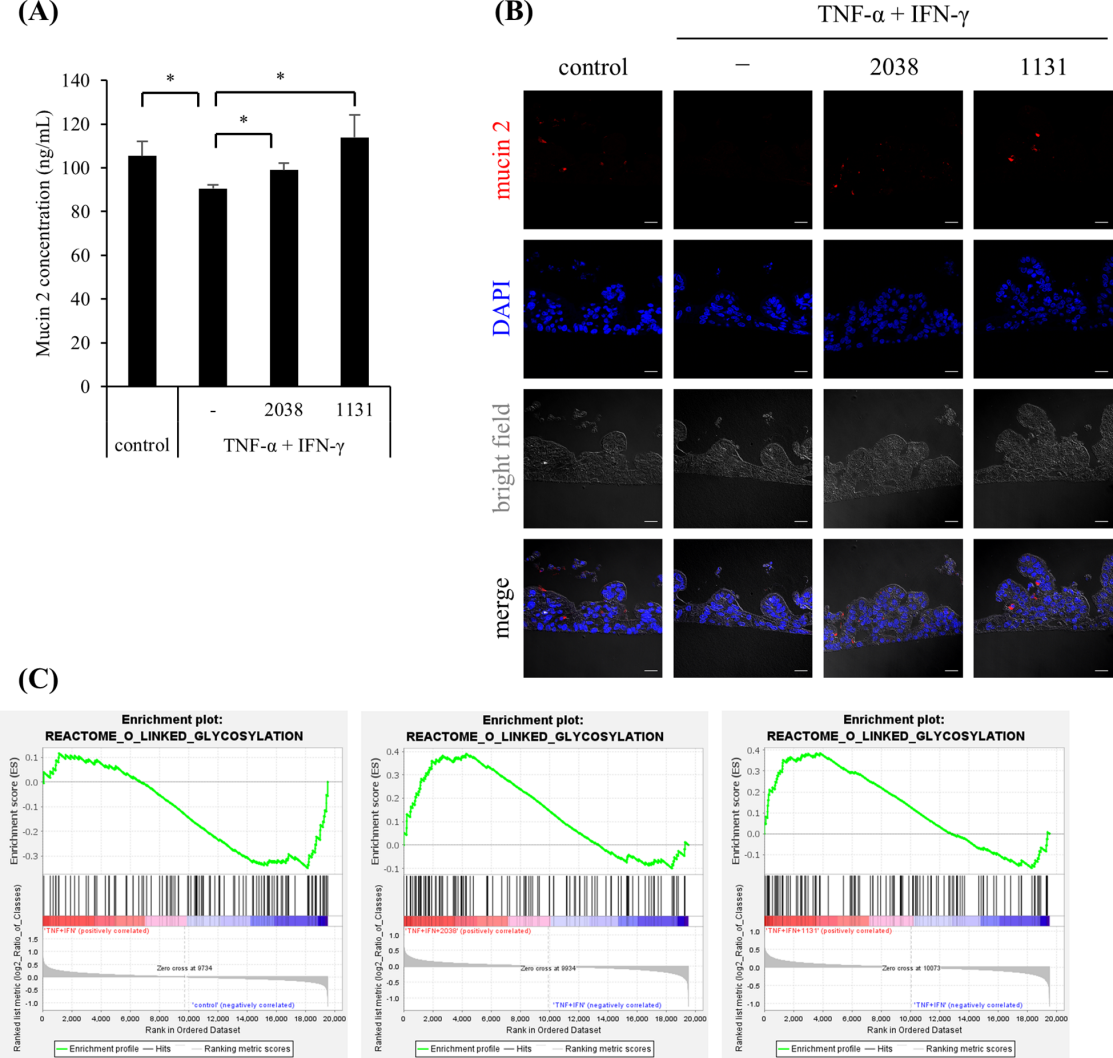
*Lactobacillus delbrueckii* subsp. *bulgaricus* 2038 and *Streptococcus thermophilus* 1131 ameliorated the decrease in mucin 2 production and mucin 2-positive cells by TNF-α and IFN-γ*. L. bulgaricus* 2038, *S. thermophilus* 1131, TNF-α, and IFN-γ were added to the cells. **(A)** Cells were collected after 42 h in the experiments. The mucin 2 concentration was measured by enzyme-linked immunosorbent assay (n = 5). Comparisons were performed with the Brunner-Munzel test followed by the Benjamini-Hochberg correction. * *P*< 0.05. **(B)** Mucin 2-positive cells were detected by immunofluorescence microscopy (scale bar, 20 μm). **(C)** Gene set enrichment analysis was performed. Enrichment plots for the gene set of REACTOME_O_LINKED_GLYCOSYLATION are shown. Comparisons between the control and TNF-α + IFN-γ groups (left panel), the TNF-α + IFN-γ and TNF-α + IFN-γ + *L*. *bulgaricus* 2038 groups (central panel), and the TNF-α + IFN-γ and TNF-α + IFN-γ + *S. thermophilus* 1131 groups (right panel) are also shown.

*MUC2* mRNA expression was increased by *L. bulgaricus* 2038 and *S. thermophilus* 1131, although the differences were not statistically significant. To evaluate this, we measured the production of mucin 2, which is mainly secreted in the small intestine and is an important component of the physical barrier. The result showed that TNF-α and IFN-γ significantly decreased the production of mucin 2 compared with that in the control group, and the treatment with both strains significantly canceled this effect (**Figure 6A**). This observation suggested that the treatments influenced the differentiation of goblet cells. Hence, we examined the number of goblet cells using immunofluorescence. TNF-α and IFN-γ markedly decreased the number of mucin 2-positive cells, whereas the treatment of both strains increased the number of these cells (**Figure 6B**).

According to the GSEA results, TNF-α and IFN-γ stimulation negatively enriched the gene set “O-linked glycosylation” in Reactome (NES = –1.48, *P* = 0.0075, *q* = 0.75), while both strains suppressed it (*L. bulgaricus* 2038; NES = 1.55, *P* = 0.0, *q* = 1.0, *S. thermophilus* 1131; NES = 1.48, *P* = 0.014, *q* = 0.93) (**Figure 6C**).

### 3.6 Microarray analysis of LAB activity

Transcriptome analysis was performed to reveal the mechanism underlying the activities of *L. bulgaricus* 2038 and *S. thermophilus* 1131 on differentiation using a microarray. For pathway analysis using MetaCore, genes with |log_2_ fold change| > 0.5 were extracted and pathway analysis was performed using DEGs. The DEGs were enriched in the gene sets described in **Table 2**. The results suggest that while TNF-α and IFN-γ induced the immune response, apoptosis, and oxidative stress, LAB suppressed the inflammatory reaction (**Table 2**).

**Figure 7:**
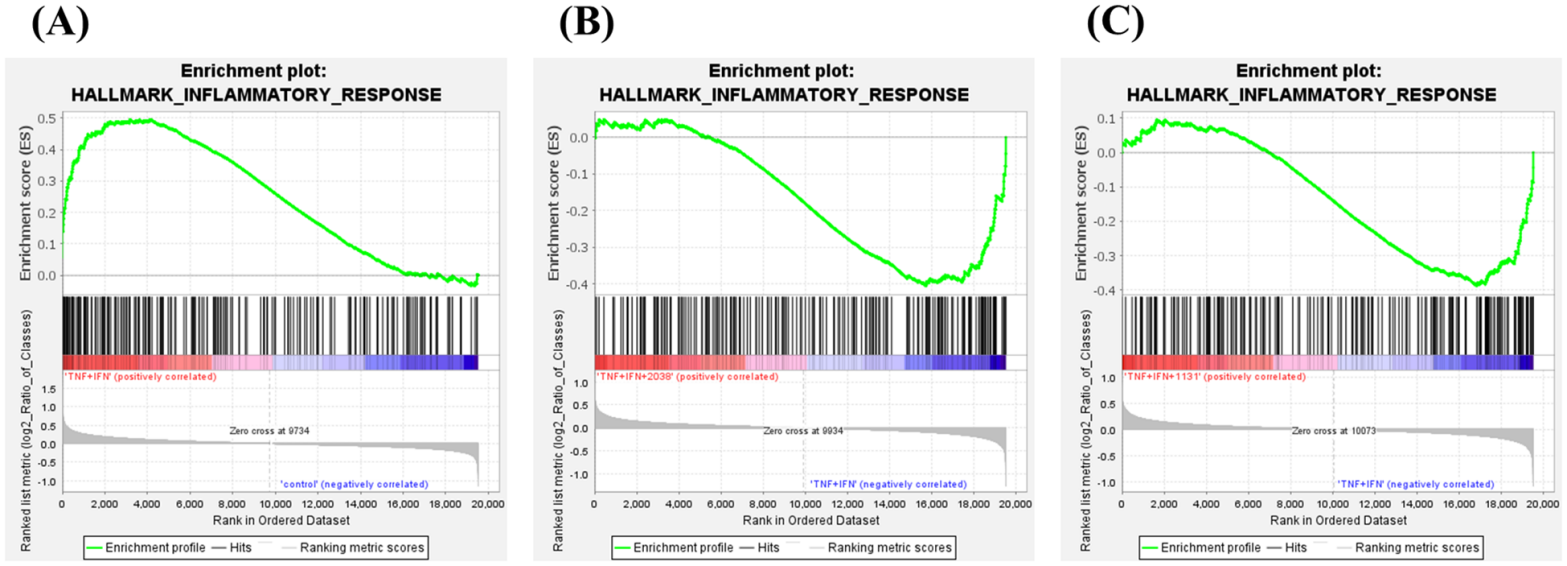
*Lactobacillus delbrueckii* subsp. *bulgaricus* 2038 and *Streptococcus thermophilus* 1131 activated and suppressed various gene sets. Gene set enrichment analysis was also performed. Enrichment plots for the gene sets of HALLMARK_INFLAMMATORY_RESPONSE are shown. Comparisons between **(A)** the control and TNF-α + IFN-γ groups, **(B)** the TNF-α + IFN-γ and TNF-α + IFN-γ + *L*. *bulgaricus* 2038 groups, and **(C)** the TNF-α + IFN-γ and TNF-α + IFN-γ + *S. thermophilus* 1131 groups are also shown.

GSEA was performed to explore differentially expressed gene sets. TNF-α and IFN-γ stimulation significantly enriched the “inflammatory response” gene set in hallmark (NES = 1.99, *P* = 0.0, *q* = 0.0), and both strains suppressed it (*L. bulgaricus* 2038; NES = –1.66, *P* = 0.0, *q* = 0.019, *S. thermophilus* 1131; NES = –1.62, *P* = 0.0, *q* = 0.012) (**Figure 7**).

## 4 Discussion

The hiPSC-SI model, in addition to being composed of the different cell types that constitute the small intestine, has the characteristic advantage of a monolayer crypt-villus structure similar to the one observed in living organisms. Moreover, regarding the barrier function, TEER value of hiPSC-SI is low compared to cell lines such as Caco-2 cells and similar to that of the small intestine of living organisms, presumably because it contains ISCs and immature progenitor cells with weak intercellular adhesion. In a recent study using hiPSC-SI, acetylsalicylic acid (ASA), a nonsteroidal anti-inflammatory drug, resulted in increased permeability and a reduction of the expression of cell markers such as *LGR5* and *MUC2* (17). The authors noted that ASA-induced cell injury causes ISC dysfunction, leading to abnormal cell differentiation. Moreover, Irsogladine, a gastroprotective drug, suppresses the increased permeability induced by ASA (17). Thus, hiPSC-SI can be used as a small intestine model that responds to stimuli in a similar manner as living organisms. However, an assay model for the co-culture of hiPSC-SIs and LAB and an experimental model to assess the impact of LAB on barrier function have yet to be established. In this study, we first examined media for use in the co-culture of LAB and hiPSC-SIs. *L. bulgaricus* 2038 was found to decrease while *S. thermophilus* 1131 markedly increased in a PS (–)-conditioned medium. This contrasting result was expected due to the differing nutritional requirements of both strains, wherein components containing differentiation factors may have affected the growth of *L. bulgaricus* 2038. Although the precise causes are not known, it is suggested that the use of conditioned medium is not suitable for co-culture. Next, we examined HBSS Glu (+) and HBSS Glu (–), which are low in nutrients and allow bacteria to remain viable. The survival rates of both strains were better in HBSS Glu (–) than HBSS Glu (+). In addition, HBSS did not alter the 3D structure, TEER value, or intestine-associated gene expression in hiPSC-SIs. hiPSC-SI did not appear confluent, but this observation may have been because the sunken crypt is not visible in the shadows shown in **Figure 3A**. Based on these findings, HBSS Glu (–) was selected as the culture medium for LAB.

Increased intestinal permeability, known as “leaky gut,” facilitates the flux of harmful substances into the blood via the intestine (25). These pathogens and toxins translocate to various organs via blood vessels, resulting in chronic inflammation and various diseases (25). Therefore, improving and maintaining a healthy intestinal barrier are important for improving overall health. In the present study, TNF-α and IFN-γ were used to simulate intestinal barrier dysfunction since they exhibit increased expression levels in patients with inflammatory bowel disease (26, 27). The levels of these cytokines are also increased in Crohn’s disease (CD), in which inflammation occurs in the digestive tract containing the small intestine, and correlates with disease activity (28–30). Therefore, the model established herein is appropriate for use as an *in vitro* leaky gut model.

In this study, although the TEER value was not changed by TNF-α and IFN-γ in hiPSC-SI, FD-4 permeability was significantly increased. Although both values are widely used as barrier indicators, the TEER value is influenced by the expression of leaky-type TJ proteins, such as CLDN-2, which contribute to the paracellular permeation of small ions (31). Hence, FD-4 permeability reflects the actual intestinal leakage in the living body. This suggests that intestinal barrier function is undermined by TNF-α and IFN-γ in hiPSC-SI. In a previous study using Caco-2 cells, cytokines were shown to increase FD-4 permeability (5). The data suggest that the responses of the intestinal barrier to TNF-α and IFN-γ are similar across the two experimental models. On the other hand, the TEER value was found to be significantly deteriorated by TNF-α and IFN-γ in experiments using Caco-2 cells (5). In both models, the gene expression levels of TJ proteins were decreased by TNF-α and IFN-γ (5). It is worth noting that the cytokine-induced changes in the expression of leaky-type TJ proteins were different and may have affected the TEER value due to the different responses to cytokines observed across the two models.

Next, we evaluated differentiation by exploiting the characteristics of hiPSC-SIs, which have various cell types containing ISCs. However, this evaluation cannot be performed using Caco-2 cells, which are exclusively composed of enterocytes. TNF-α and IFN-γ significantly decreased the gene expression of *LGR5*, *VIL1*, *MUC2*, and *LYZ*, which are the marker gene of ISC, enterocyte, goblet cell, and Paneth cell, respectively. In patients with CD, *LGR5* expression and *LYZ* ^+^ cells are decreased in the ileal crypt regions (32). Moreover, crypts from TNF^ΔARE^ mice, transgenic CD-like ileitis model, failed to grow into organoids (32). These data suggest that crypt inflammation leads to a loss of stemness. Enrichment analysis showed that TNF-α and IFN-γ significantly increased the expression of the gene set related to inflammatory response. This suggests that TNF-α and IFN-γ damage ISCs and influence subsequent cell differentiation and proliferation.

In the present study, we evaluated the effects of *L. bulgaricus* 2038 and *S. thermophilus* 1131 on hiPSC-SI. First, with regards to their effects on barrier function, both strains ameliorated FD-4 permeability deteriorated by TNF-α and IFN-γ. These results were similar to those of previous experiments using Caco-2 cells (5). As hiPSC-SI is a better biological model than Caco-2 cells, this study supports the hypothesis that the intake of both strains is effective for treating leaky guts. Moreover, both strains ameliorated the decreased expression of TJ proteins by TNF-α and IFN-γ in hiPSC-SI. This observation suggests that both strains ameliorate intestinal barrier dysfunction by modulating TJ protein expression. We previously reported that both strains activated AMP-activated protein kinase (AMPK) which is involved in TJ expression (5). Metformin (33), an AMPK activator, and food materials (34, 35) increase TJ expression in Caco-2 cells. Furthermore, ZO-1 expression in the jejunum was reduced in AMPK VilCre knockout mice (36). These observations suggest that *L. bulgaricus* 2038 and *S. thermophilus* 1131 increase TJ expression by activating AMPK in hiPSC-SI. To verify this hypothesis, we will confirm that the effect of amelioration of intestinal barrier by both strains is canceled by an AMPK inhibitor in the experiments using hiPSC-SI.

In terms of differentiation, *L. bulgaricus* 2038 and *S. thermophilus* 1131 ameliorated the decreased expression of the marker genes by TNF-α and IFN-γ. Enrichment analysis showed that both strains significantly ameliorated the expression of the gene set related to the inflammatory response increased by TNF-α and IFN-γ. LAB have been reported to modulate inflammatory responses in IECs. *Lactobacillus delbrueckii* exerts anti-inflammatory activity in porcine IECs via Toll-like receptor (TLR) 2 signaling (37). Components such as peptidoglycan and lipoteichoic acid in the cell wall of Gram-positive bacteria are also recognized by TLR2 (38, 39). This highlights the potential that both strains act as anti-inflammatory factors in ISCs via TLR2 activation. This hypothesis can also be verified by the experiment using an inhibitor of TLR2.

In the small intestine, mucin 2 is mainly expressed and secreted from goblet cells as a gel-forming mucin (40). The mucus layer acts as a physical barrier and contains antimicrobial peptides that keep harmful substances, including pathogens, away from the intestinal epithelial cells (41). Stimulation of TNF-α and IFN-γ significantly decreased the production of mucin 2, and the addition of *L. bulgaricus* 2038 and *S. thermophilus* 1131 significantly suppressed it. Immunofluorescence microscopy analysis indicated that both strains improved the decreased number of goblet cells by TNF-α and IFN-γ. This suggests that the anti-inflammatory effects of *L. bulgaricus* 2038 and *S. thermophilus* 1131 on ISCs ameliorate cell differentiation. In contrast, LAB has been reported to directly stimulate goblet cells and promote the production of mucin 2. Lipoteichoic acid derived from the cell wall of *Lactobacillus paracasei* has been reported to promote the production of mucin 2 via TLR2 stimulation in CMT93 cells, a mouse intestinal goblet cell line (42). This suggests that *L. bulgaricus* 2038 and *S. thermophilus* 1131 have the potential to promote mucin 2 production via recognition by TLR2 expressed on the surface of goblet cells.

GSEA showed that both strains improved the decreased expression of the gene set of O-linked glycosylation by TNF-α and IFN-γ. As previously reported, more than 80% of mucin 2 is constituted by O-glycans (43, 44). Hence, O-linked glycosylation is necessary for biosynthesis of mucin 2. This observation may be attributed to increases in goblet cells by both strains, mediating cell differentiation via anti-inflammatory activity on stem cells or directly via the activity of both strains on goblet cells through TLR2.

One of the limitations of this study is that it is unclear in which cells inflammation occurs by TNF-α and IFN-γ. We hypothesize that TNF-α and IFN-γ induce inflammation in stem cells, leading to abnormal differentiation, which is suppressed by the anti-inflammatory effects of LAB, but the details are unknown. To elucidate the mechanism, it would be useful to analyze the expression of inflammation associated genes in stem cells by sorting LGR5^+^ cells or by using single cell RNA-Seq (scRNA-Seq). scRNA-Seq would reveal the expression profile in each cell type, which would provide important data for mechanistic analysis.

To summarize, this study established an experimental model of hiPSC-SI stimulated by TNF-α and IFN-γ to assess their effects on barrier function when co-culturing hiPSC-SI and LAB. In this model, *L. bulgaricus* 2038 and *S. thermophilus* 1131 were found to suppress cytokine-induced intestinal barrier dysfunction by modulating the expression of TJ-associated proteins. Furthermore, both strains were found to ameliorate the aberration of cell differentiation by TNF-α and IFN-γ via anti-inflammatory effect on ISCs. Since differentiation experiments are not feasible in cell lines, such as Caco-2 cells, the hiPSC-SI model provides a distinct advantage over existing models. This model highlighted the fact that both *L. bulgaricus* 2038 and *S. thermophilus* 1131 could induce the production of mucin 2 and increase the number of goblet cells, indicative of a strengthening of the intestinal barrier. Taken together, these results demonstrate that these strains can ameliorate intestinal barrier integrity and homeostasis disrupted by TNF-α and IFN-γ.

## Data Availability

The datasets presented in this study can be found in online repositories. The names of the repository/repositories and accession number(s) can be found below: GSE291253 (GEO).
