# Supplementary figures and images for: Lactobacillus delbrueckii subsp. bulgaricus 2038 and Streptococcus thermophilus 1131 ameliorate barrier dysfunction in human induced pluripotent stem cell-derived crypt-villus structural small intestine
Source: Front Immunol. 2025 Jun 11;16:1585007. doi: 10.3389/fimmu.2025.1585007 (PMC12190435; doi:10.3389/fimmu.2025.1585007)

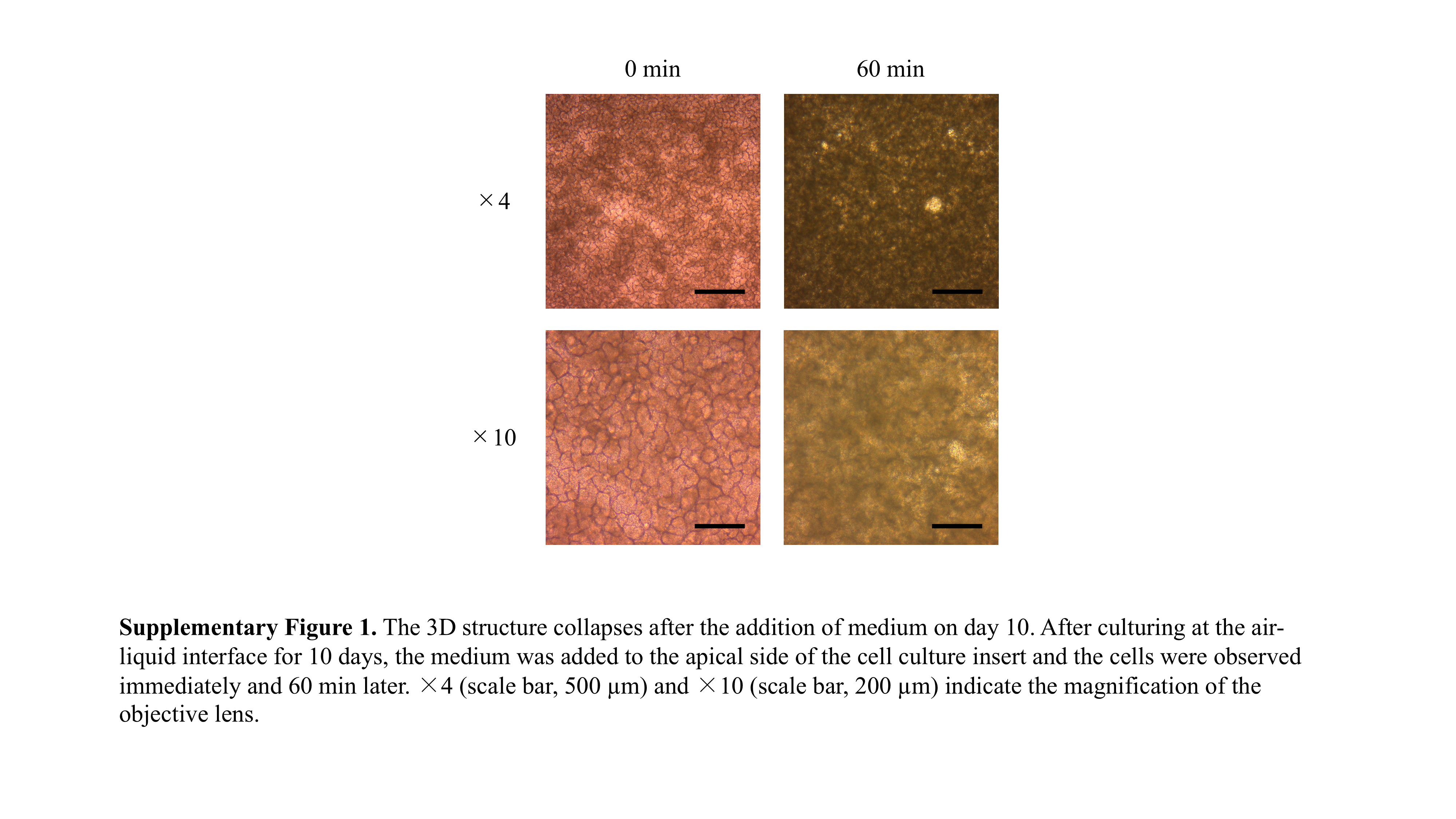

Supplement: Supplementary file 1 [file Image1.tif]

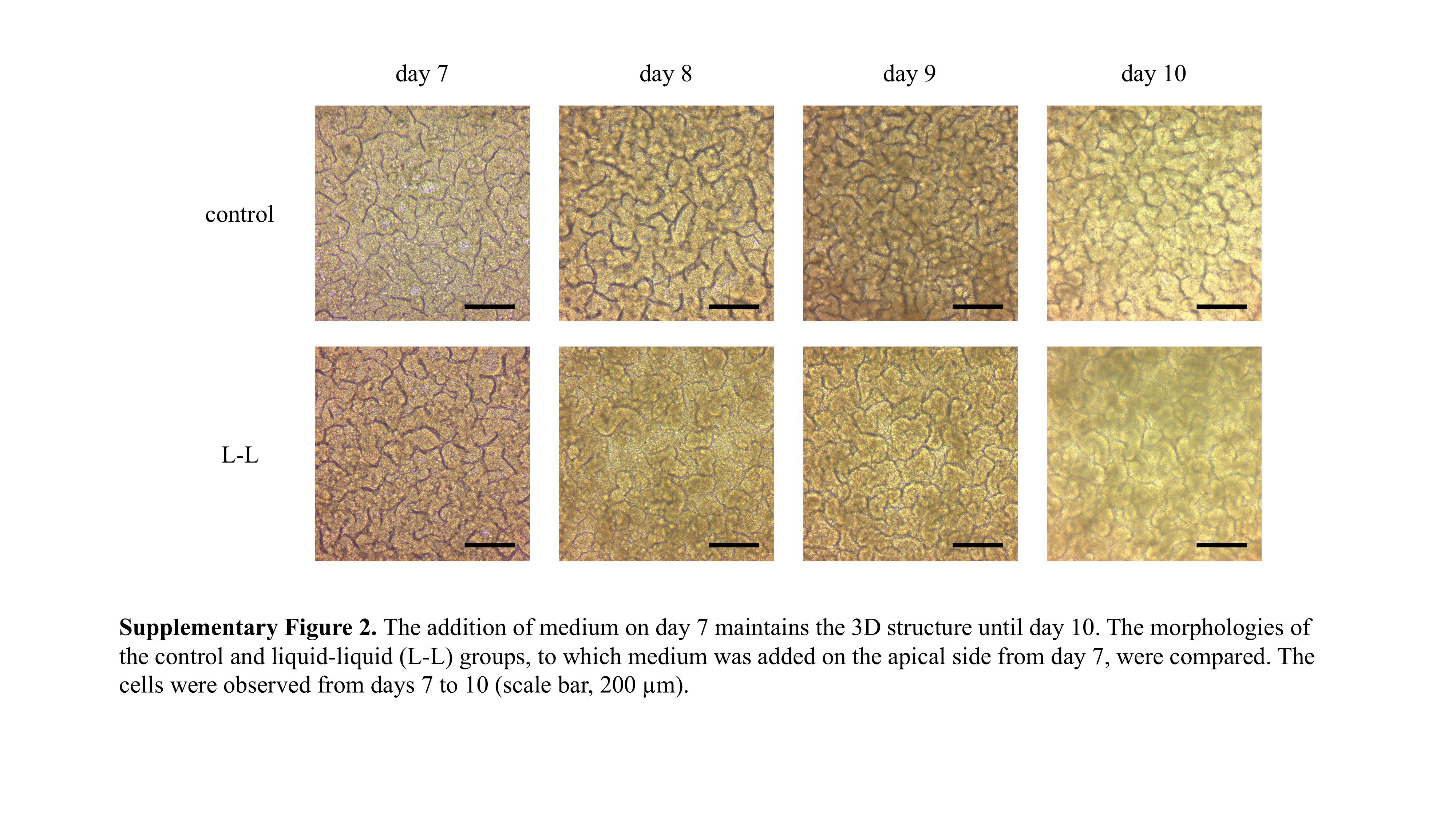

Supplement: Supplementary file 2 [file Image2.tif]

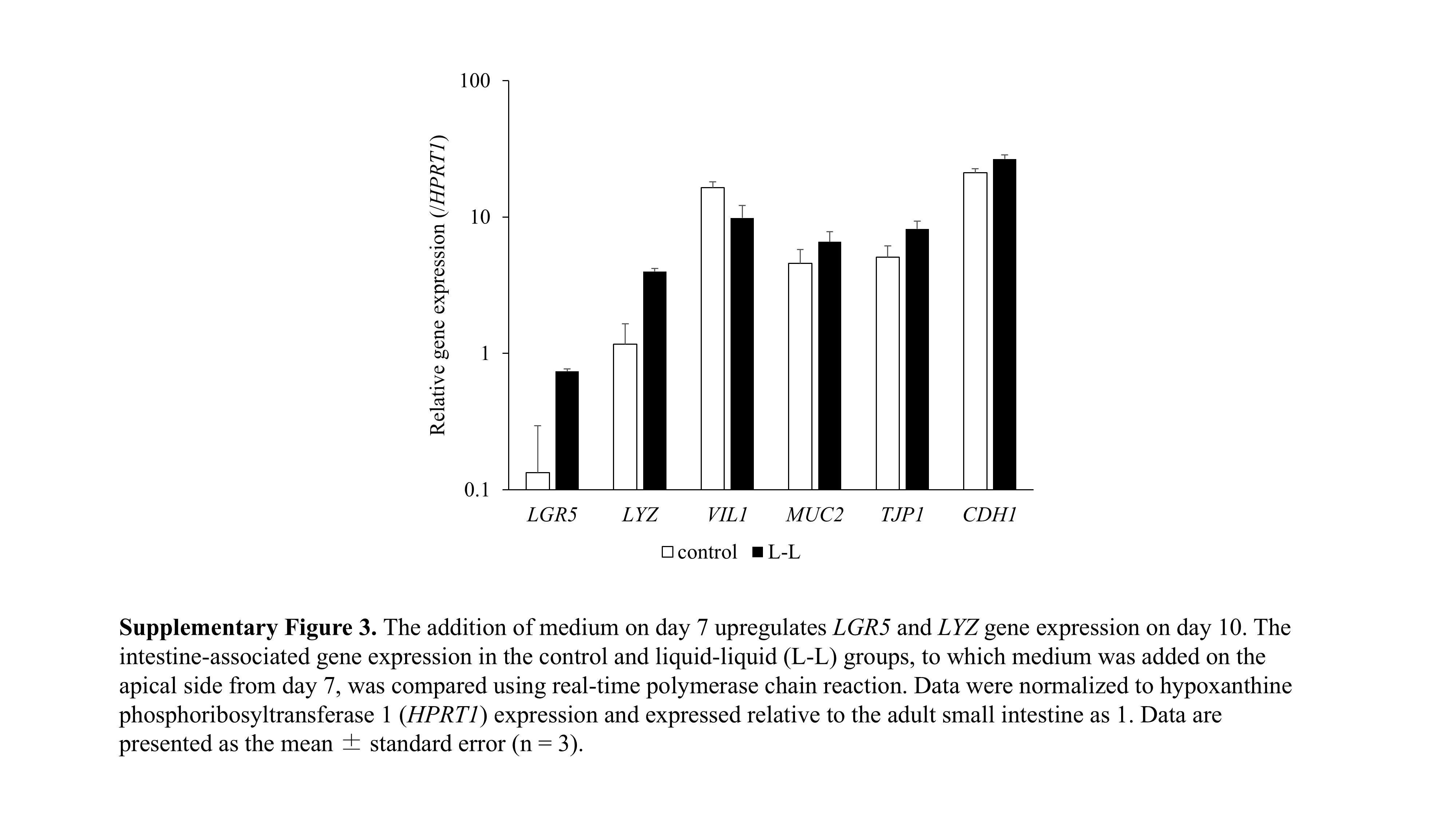

Supplement: Supplementary file 3 [file Image3.tif]

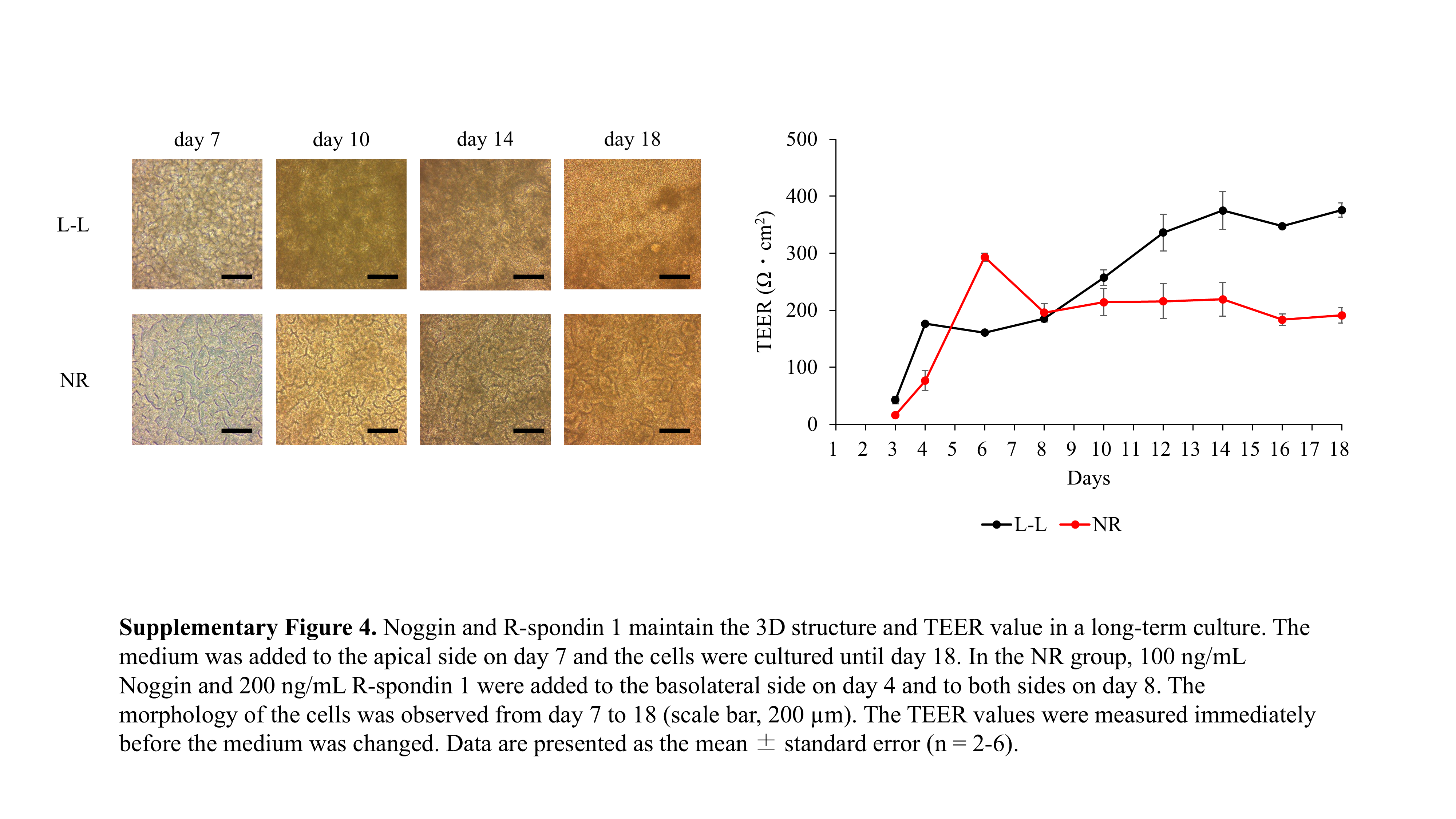

Supplement: Supplementary file 4 [file Image4.tif]

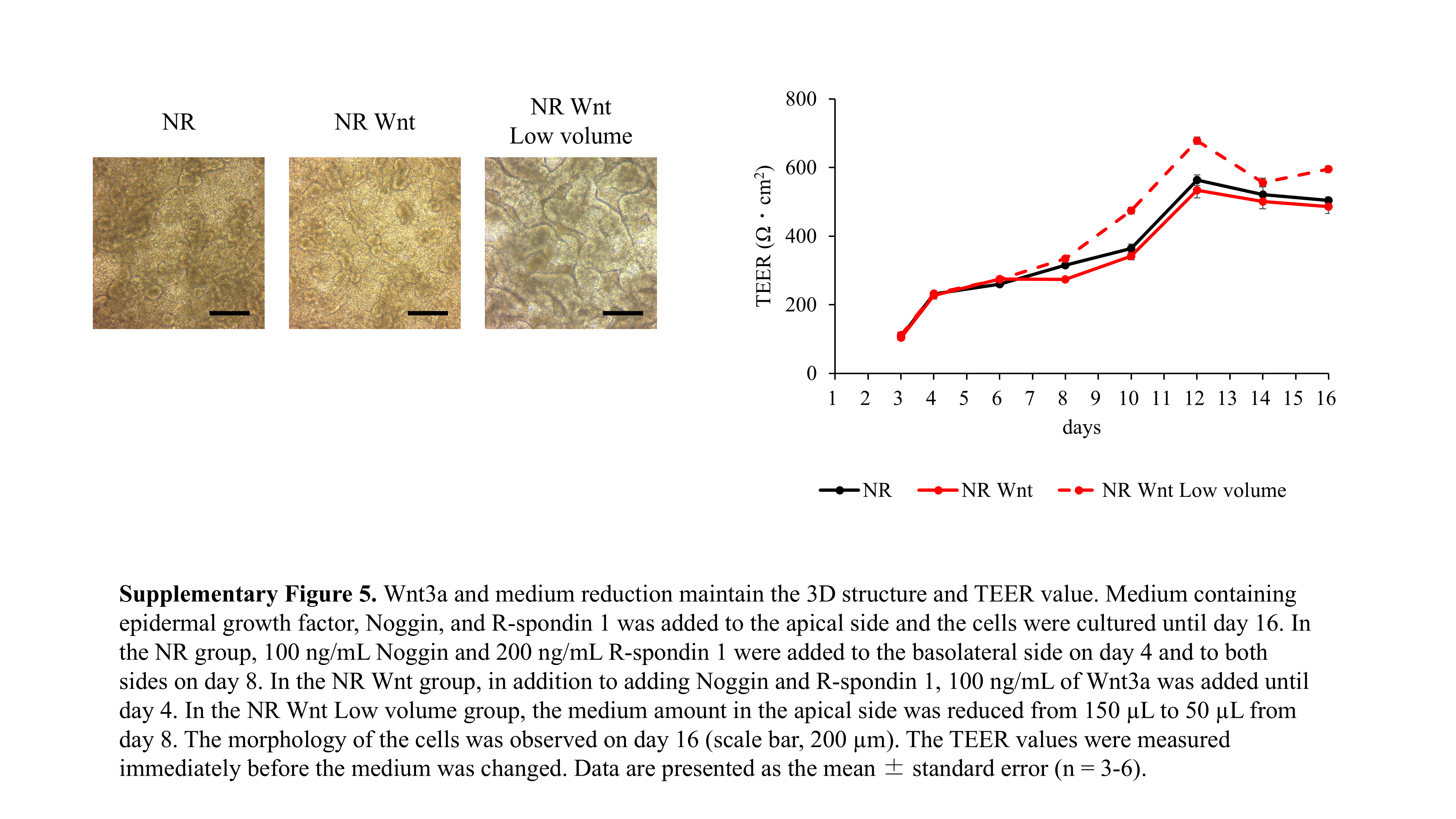

Supplement: Supplementary file 5 [file Image5.tif]

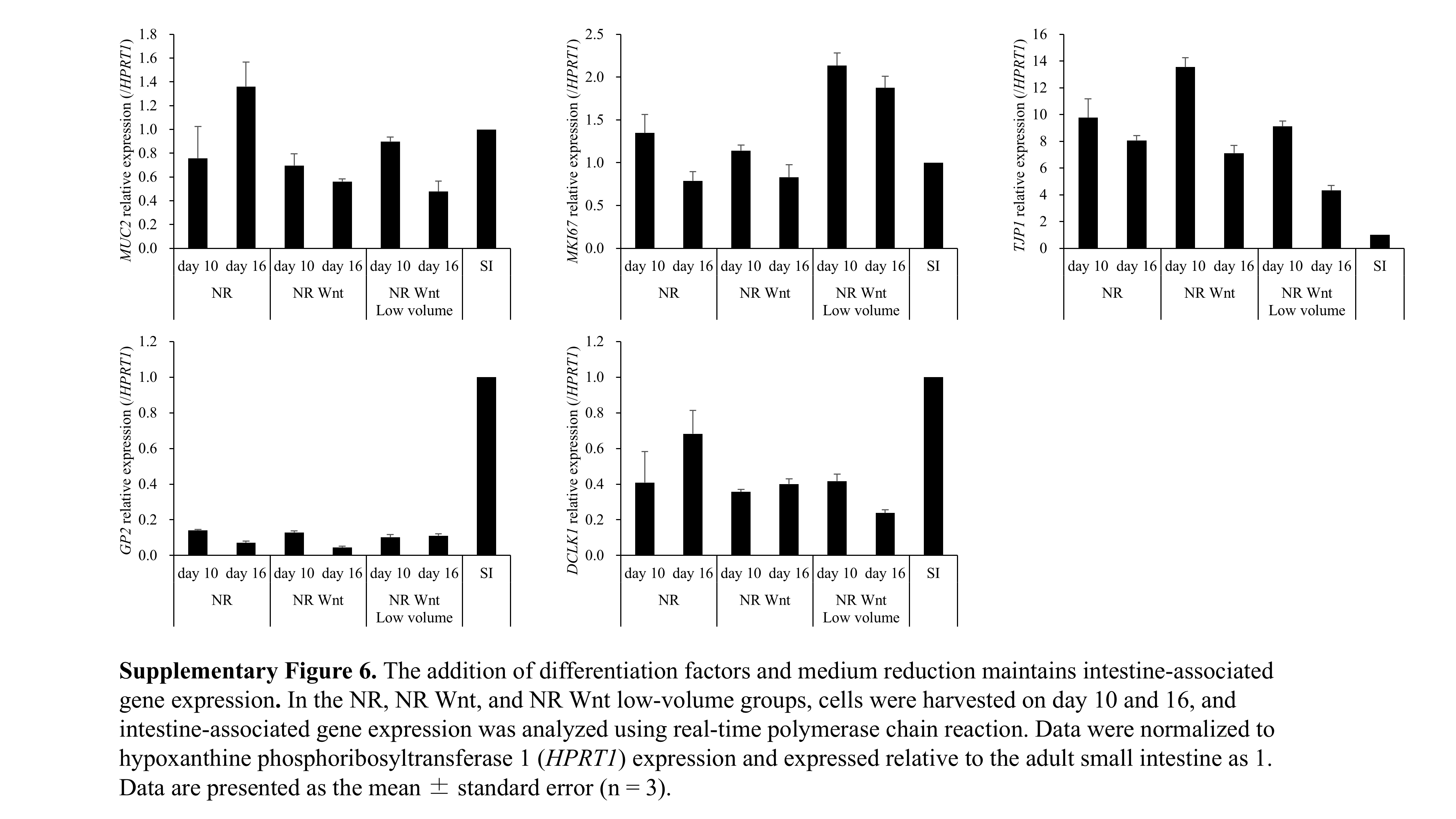

Supplement: Supplementary file 6 [file Image6.tif]
