# Supplementary material for: Lactobacillus delbrueckii subsp. bulgaricus 2038 and Streptococcus thermophilus 1131 ameliorate barrier dysfunction in human induced pluripotent stem cell-derived crypt-villus structural small intestine
Source: Front Immunol. 2025 Jun 11;16:1585007. doi: 10.3389/fimmu.2025.1585007 (PMC12190435; doi:10.3389/fimmu.2025.1585007)
Supplement: Supplementary file 7 [file SupplementaryFile1.docx]

Supplementary Material

In a previous study, we established a method for culturing human intestinal organoids (HIOs) and one for culturing the HIOs on cell culture inserts to construct a model for a human induced pluripotent stem cell-derived crypt-villus structural small intestine (hiPSC-SI). However, exposing bacteria remained a challenge because the medium on the apical side was removed on day 3 of culture at the air-liquid interface. Moreover, the three-dimensional (3D) structure collapsed when the medium was added again from day 10, when the cells matured and could be used as an evaluation system (Supplementary Figure 1). Thus, there was a need to establish a method by which to maintain the crypt villi-like structure, even with the medium in the insert. To this end, the control and liquid-liquid (L-L) groups, to which medium was added from days 10 and 7, respectively, were compared. Although changes in the 3D structure were observed after 3 and 6 h in the control group, the 3D structure was maintained until day 10 in the L-L group (Supplementary Figure 2). The results of gene expression analysis showed that on day 10, the increase in *LGR5* (intestinal stem cells) and *LYZ* (Paneth cells) in the L-L group, and other genes of intestinal markers (*VIL1* enterocyte, *MUC2*; goblet cell; *TJP1*, tight junction; and *ECAD*, cell adhesion) did not change (Supplementary Figure 3). Furthermore, in long-term culture, the L-L group lost its 3D structure after day 14, whereas the addition of Noggin and R-spondin 1 to the basolateral side after day 4 and to both sides after day 8 maintained the 3D structure until day 18, and the trans-epithelial electrical resistance (TEER) value remained constant (Supplementary Figure 4). Additional studies showed that medium addition from day 8 was optimal for maintaining the 3D structure, indicating that the medium should be added from day 8 (data not shown). Moreover, the addition of Wnt3a up to day 4 and a reduction in the amount of medium in the apical side from 150 µL to 50 µL from day 8 maintained the 3D structure, with no abnormalities observed in the TEER values until day 16 (Supplementary Figure 5). Gene expression analysis showed that the expression of *MUC2* (goblet cells), *MKI67* (cell proliferation), and *TJP1* (tight junctions) was sufficient, decreasing only slightly on day 16 compared to that on day 10. In addition, the expression of *GP2* (M cells responsible for bacterial uptake) and *DCLK1* (tuft cells responsible for the parasite infection response) was observed at lower levels than in the adult small intestine (Supplementary Figure 6). The hiPSC-SI culture protocol based on the above experiments is shown in Figure 1.
